# Circulating amino acids and acylcarnitines correlated with different CAC score ranges in diabetic postmenopausal women using LC–MS/MS based metabolomics approach

**DOI:** 10.1186/s12902-022-01073-9

**Published:** 2022-07-21

**Authors:** Shaghayegh Hosseinkhani, Pooneh Salari, Fatemeh Bandarian, Mojgan Asadi, Shapour Shirani, Niloufar Najjar, Hojat Dehghanbanadaki, Parvin Pasalar, Farideh Razi

**Affiliations:** 1 Metabolomics and Genomics Research Center, Endocrinology and Metabolism Molecular-Cellular Sciences Institute, Tehran University of Medical Sciences, Tehran, Iran; 2 Department of Clinical Biochemistry, Faculty of Medicine, Tehran University of Medical Sciences, Tehran, Iran; 3 Medical Ethics and History of Medicine Research Center, Tehran University of Medical Sciences, Tehran, Iran; 4 Endocrinology and Metabolism Research Center, Endocrinology and Metabolism Clinical Sciences Institute, Tehran University of Medical Sciences, Tehran, Iran; 5 Osteoporosis Research Center, Endocrinology and Metabolism Clinical Sciences Institute, Tehran University of Medical Sciences, Tehran, Iran; 6 Imaging Department, Tehran Heart Center, Tehran University of Medical Sciences, Tehran, Iran; 7 Metabolic Disorders Research Center, Endocrinology and Metabolism Molecular-Cellular Sciences Institute, Tehran University of Medical Sciences, Tehran, Iran; 8 Diabetes Research Center, Endocrinology and Metabolism Clinical Sciences Institute, Tehran University of Medical Sciences, Tehran, Iran

**Keywords:** Metabolites, Acylcarnitine, Amino acid, Cardiovascular disease, Diabetes mellitus, Postmenopausal women

## Abstract

**Background:**

Diabetes mellitus (DM) and its cardiovascular disease (CVD) complication are among the most frequent causes of death worldwide. However, the metabolites linking up diabetes and CVD are less understood. In this study, we aimed to evaluate serum acylcarnitines and amino acids in postmenopausal women suffering from diabetes with different severity of CVD and compared them with healthy controls.

**Methods:**

Through a cross-sectional study, samples were collected from postmenopausal women without diabetes and CVD as controls (*n* = 20), patients with diabetes and without CVD (*n* = 16), diabetes with low risk of CVD (*n* = 11), and diabetes with a high risk of CVD (*n* = 21) referred for CT angiography for any reason. Metabolites were detected by a targeted approach using LC–MS/MS and metabolic -alterations were assessed by applying multivariate statistical analysis. The diagnostic ability of discovered metabolites based on multivariate statistical analysis was evaluated by ROC curve analysis.

**Results:**

The study included women aged from 50–80 years with 5–30 years of menopause. The relative concentration of C14:1, C14:2, C16:1, C18:1, and C18:2OH acylcarnitines decreased and C18 acylcarnitine and serine increased in diabetic patients compared to control. Besides, C16:1 and C18:2OH acylcarnitines increased in high-risk CVD diabetic patients compared to no CVD risk diabetic patients.

**Conclusion:**

Dysregulation of serum acylcarnitines and amino acids profile correlated with different CAC score ranges in diabetic postmenopausal women. (Ethic approval No: IR.TUMS.EMRI.REC.1399.062).

**Supplementary Information:**

The online version contains supplementary material available at 10.1186/s12902-022-01073-9.

## Background

Metabolomics approaches have become a capable method for the identification of biomarkers for screening, diagnosis, and monitoring of various diseases in the last decades. The concept of metabolomics discloses the downstream of genome, epigenome, transcriptome, and proteome and is known as a reflection of an existing phenotype in systems biology [1–3]. Assessment of metabolite profiles such as acylcarnitine metabolites [4], branched-chain and aromatic amino acids [5] could help to find novel markers for predicting DM and the risk of CVD in DM [6, 7, 8].

Evidence suggests that the risk of CVD events in women with DM is stronger and more age-related than diabetic men [9, 10], also this association was noticed to be potent in postmenopausal women rather than the premenopausal one [11]. In essence, menopause is gone together with fluctuations in body mass index, distribution of adipose tissue, and energy disbursement, along with insulin secretion and sensitivity that can affect the development of DM [12, 13].

In the present study, we applied a high throughput technique, LC–MS/MS-based metabolomics approach, to analyze amino acids and acylcarnitine metabolites in postmenopausal women suffering from DM with different severity of CVD compared with the control group to take a step toward discovering novel and credible metabolite.

## Research design and methods

### Study populations and classification of participants

This cross-sectional study was carried out from April 2019 until December 2020 on postmenopausal women who had been referred to Tehran heart hospital and underwent coronary CT scans (Dual Source Flash-128 slice) for any reason. Coronary artery calcium score (CACS) values which can be detected by cardiac CT data were calculated for them by an expert radiologist based on the Agatston Score quantification method. A CACS reflects the atherosclerotic coronary artery disease (ACAD) burden and determines future CVD risk [14].

Participants were divided into three groups based on the CACS test. 1) no CACS: A score of zero indicating there is no risk of coronary heart disease, 2) low CACS: A score between 1 to 100, with low to the mild risk of heart disease and 3) high CACS: A score greater than 100, which point to clogged arteries and is associated with a relatively higher risk of heart diseases over the next years [15]. Diabetic patients were identified according to their medical history.

All participants had neither acute infection, history of cancer, renal, gastrointestinal nor thyroid diseases. Also, patients who were taking certain medications such as hormones, corticosteroids, heparin, and thyroid hormones were excluded.

Finally, the study comprised 68 postmenopausal women distributed into four study groups: (1) 16 DM patients without CVD; (2) 11 DM patients with low-risk CVD; (3) 21 DM patients with high-risk CVD; (4) 20 non-diabetic subjects with CACS of zero as controls.

### Blood sampling and biochemical analysis

A venous blood sample was obtained from patients and stored at -80 °C until the analysis. Information on demographics and medical status was captured by questionnaires. Biochemical analytes were measured by COBAS c311 (Roche Diagnostics).

### Sample preparation and metabolite quantification

The metabolomics assessment method had been described in detail in the previous study [16]. Briefly, the serum samples metabolomics measurements were performed using flow injection analysis on MS/MS technology, an AB Sciex 3200 triple quadruple system. The ion source was electrospray ionization (ESI). Multiple reaction monitoring (MRM) with positive ion mode was performed to scan analytes. The mobile phase which transferred the components to be assessed was acetonitrile aqueous solution. Isotope-labeled internal standards of amino acids and acylcarnitines were used for targeted quantification. Data acquisition and analysis were accomplished on Multiquant 3.0.2 software. The quality control samples were analyzed together with the samples in each run. The mean of estimated inter-assay precision (reported as a coefficient variation) for AAs and acylcarnitines were less than 8.7% and 12.3%, the estimated mean bias was below 8.8% and 10.2% respectively.

### Statistical analysis

#### Data preprocessing

The missing values were replaced by the default method of Metabo-Analyst (half of the minimum positive values detected in the data). Different types of data normalization, transformation, and scaling were assessed to make samples and metabolite concentrations more comparable and transform the data into a better Gaussian-type distribution [17]. Finally, the cube root transformation and Pareto scaling was performed.

#### Discovering the metabolites changes

Determining the significant differences among experimental groups from complex mass spectrometry data was carried out based on univariate and multivariate statistical analysis. First of all, based on the normality results of the Kolmogorov Smirnov test, the one-way ANOVA analysis with Tukey’s HSD test was conducted to determine the significant metabolites. Logistic regression was performed to adjust the effect of age, BMI, time of menopause, lipid profile (cholesterol, HDL-C, LDL-C), diabetes duration, and medications (B-blockers, metformin, statin, anticoagulants, and nitroglycerin) on each metabolite.

Partial least squares discriminant analysis (PLS-DA) was used to distinguish and visualize the metabolites which are responsible for the discrepancy between the groups. Afterward, the variable importance in the project (VIP) value that was generated in PLS-DA processing was applied for selection of discriminating metabolites. VIP score is a weighted sum of squares of the PLS that accounting the degree of Y-variation in each dimension. In this study, variables with *P*-value less than 0.05 based on ANOVA and VIP values higher than one were considered as discriminating metabolites [18, 19] and analyzed by logistic regression to measure their associations with CVD development. To validate the robustness of the PLS-DA model and to assess the amount of overfitting, permutation tests with 100 iterations were performed. The multivariable analysis was performed by the backward method selection procedure to assess the effect of the confounder. To more clearly characterize the profile of metabolites, a plot based on the Pearson correlation coefficient was used.

The procedure was carried out using Metabo-analyst software version 5.0 (https://www.metaboanalyst.ca) and IBM SPSS Statistics software version 26 (https://www.ibm.com/analytics/spss-statistics-software).

## Results

### Baseline characteristics of the study population

Demographic information and clinical characteristics of the study groups (DM patients without CVD (16); DM patients with low-risk CVD (11); DM patients with high-risk CVD (21); non-diabetic subjects with CACS of zero as controls (20)) are summarized in Table 1. Briefly, the study included women with a mean of 63.9 years of age, 29.8 kg/m^2^ of BMI, and 5–30 years of menopause. There was no significant difference between the three diabetes groups in terms of the duration of diabetes. Also, this trend is similar for HbA1c, which could be because patients were under-control and received proper medication.

**Table 1:** Demographic and clinical characteristics of participants

	Controls	Diabetes without CVD	Diabetes + Low-risk CVD	Diabetes + High-risk CVD	*P*-value
**N (%)**	20 (29.4%)	16 (23.5%)	11 (16.2%)	21 (30.9%)	
**Age (year)**	62.55 ± 5.708	62.06 ± 6.082	64.55 ± 5.681	66.62 ± 8.255	0.145^*^
**Years of menopause**	15.4 ± 7.148	15.94 ± 9.943	15.36 ± 7.406	19.33 ± 11.702	0.513^*^
**Duration of diabetes (year)**	0	6.87 ± 3.55	16 ± 8.56	11.6 ± 7.76	≤ 0.001^*^
**BMI (kg/m**^2^)	30.37 ± 4.9529	29.075 ± 4.1130	28.227 ± 4.3941	31.671 ± 6.7291	0.291^*^
**Biochemistry tests**					
** Uric Acid (mg/dL)**	4.895 ± 1.3763	5.406 ± 1.5181	4.873 ± 1.4065	5.176 ± 1.0392	0.632^*^
** Creatinine (mg/dL)**	0.76 (0.69–0.87)	0.86 (0.75–0.93)	0.87 (0.73–1.01)	0.81 (0.71–0.88)	0.438^**^
** GFR (mL/min/1.73m**^2^)	93.5 (90–97.5)	87.5 (76–97.5)	84 (71–98)	87 (80–95)	0.516^**^
** HbA1c (%)**	6.04 (5.76–6.88)	5.74 (5.63–6.45)	6.51 (5.83–7.31)	5.72 (5.44–6.1)	0.076^**^
** Cholesterol (mg/dL)**	158.4 ± 36.151	157.19 ± 25.002	165.73 ± 22.258	179.48 ± 35.785	0.116^*^
** HDL-C (mg/dL)**	43.8 ± 11.669	41.06 ± 6.382	49 ± 16.131	39.95 ± 7.486	0.117^*^
** LDL-C (mg/dL)**	70.2 ± 31.388	76.25 ± 26.07	68.36 ± 18.101	91.71 ± 29.16	0.053^*^
** AST (U/L)**	18 (16–23.5)	20 (17–21.5)	20 (18–21)	19 (15–23)	0.757^**^
** ALT (U/L)**	10.5 (8–15.5)	8.5 (7–14.5)	12 (6–17)	10 (7–13)	0.563^**^
**Medications**					
** Aspirin**	9 (45%)	7 (43.8%)	5 (45.5%)	13 (61.9%)	0.63
** Anticonvulsant**	0	0	2 (18.2%)	1 (4.8%)	0.085
** Diuretics**	1 (5%)	1 (6.3%)	3 (27.3%)	6 (28.6%)	0.094
** B-blockers**	5 (25%)	5 (31.3%)	9 (81.8%)	15 (71.4%)	0.001
** ACEi**	1 (5%)	1 (6.3%)	0	3 (14.3%)	0.467
** ARB**	8 (40%)	9 (56.3%)	7 (63.6%)	13 (61.9%)	0.466
** CCB**	3 (15%)	0	2 (18.2%)	5 (23.8%)	0.235
** Metformin**	0	13 (81.3%)	10 (90.9%)	20 (95.2%)	< 0.001
** Insulin**	0	3 (18.8%)	2 (18.2%)	6 (28.6%)	0.096
** Statins**	11 (55%)	10 (62.5%)	6 (54.5%)	19 (90.5%)	0.057
** Anticoagulant**	2 (10%)	0	0	5 (23.8%)	0.064
** Nitroglycerine**	8 (40%)	1 (6.3%)	1 (9.1%)	14 (66.7%)	< 0.001

*BMI* Body mass index, *GFR* Glomerular filtration rate, *LDL-C* Low-density lipoprotein cholesterol, *HDL-C* High-density lipoprotein cholesterol, *ALT* Alanine aminotransferase, *AST* Aspartate aminotransferase, *ACEi* Angiotensin-converting enzyme inhibitors, *ARB* Angiotensin receptor blockers, *CCB* Calcium channel blockers

Data are represented as n (%), means ± standard deviation, median (interquartile range(Q1-Q3))

^*^P-values for comparisons between groups derived by ANOVA test

^**^P-values for comparisons between groups derived by Kruskal–Wallis’s test

*P*-values for comparisons between groups of medications derived by Pearson Chi-Square test

### Selection of discriminating metabolites

**Fig. 1:**
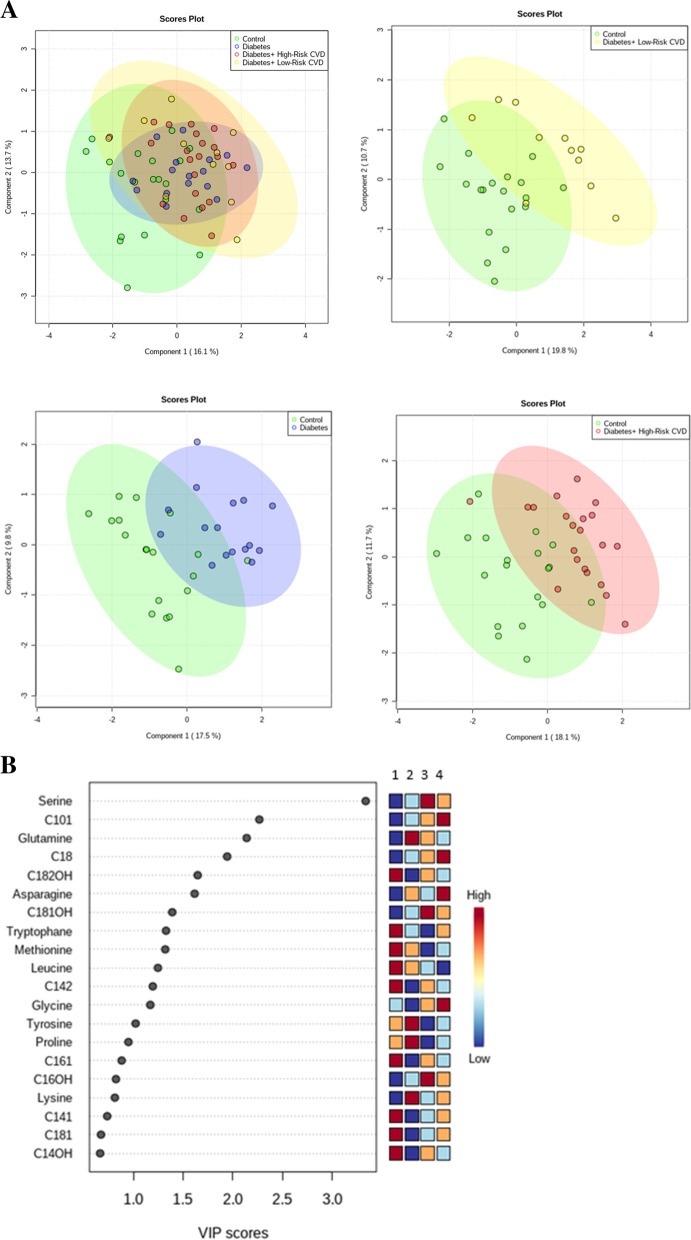
**A** Score plots of partial least squares (PLS-DA) scatter plots (green = control, blue = diabetes, yellow = diabetes + low-risk CVD, and red = diabetes + high-risk CVD) and **B** variable importance in projection (VIP) generated from PLS-DA. Metabolites with a VIP score ≥ 1 were considered as discriminating metabolites (1 = Controls, 2 = Diabetes without CVD, 3 = Diabetes + High-risk CVD, 4 = Diabetes + Low-risk CVD)

The measured concentration and coefficients of variation of each metabolite and the result of data preprocessing are represented in Supplementary Table S1, S2 and Figure S1, respectively. The PLS-DA score plots are demonstrated in Fig. 1A. Control samples were comparatively separated from patient samples (DM, DM + low-risk CVD, and DM + high-risk CVD) along with the scores for the first two components, which explained the variance (diabetes compared to control: PC1 = 17.5, PC2 = 9.8; diabetes with low-risk CVD compared to controls: PC1 = 19.8, PC2 = 10.7; diabetes with high-risk CVD compared to control: PC1 = 18.1, PC2 = 11.7). PLS-DA model validation is performed by permutation tests based on the separation distance. The *P*-value based on permutation was less than 0.01, indicating that the model was not over-fitted (supplementary Figure S2).

Important features in metabolites discrimination identified by PLS-DA are depicted by VIP score plots in Fig. 1B. The colored boxes on the right indicate the relative concentrations of the corresponding metabolite in each group under study.

Variables with a VIP ≥ 1 and *P*-value < 0.05 (based-on ANOVA analysis, Table S3) were considered to be significantly different variables and listed in Table 2.

**Table 2:** Unadjusted and adjusted odds ratio (OR) analysis for the significantly altered metabolites associated with high-risk CVD, low-risk CVD, and diabetes mellitus

**Metabolites**	**Diabetes/ controls**				**Diabetes + low risk CVD/ controls**			
	OR (95% CI)	*P*-Value	*P*-Value^a^	*P*-Value^b^	OR (95% CI)	*P*-Value	*P*-Value^a^	*P*-Value^b^
Serine	1.021 (1.003–1.039)	**0.025**	**0.026**	**0.000**	1.026 (1.006–1.045)	**0.009**	**0.009**	**0.000**
C10:1	1.003 (0.999–1.007)	0.134	0.166	0.899	1.006 (1.001–1.01)	**0.016**	**0.017**	0.579
C14:1	0.979 (0.964–0.994)	**0.007**	**0.006**	**0.000**	0.989 (0.974–1.004)	0.147	0.151	**0.000**
C14:2	0.974 (0.958–0.991)	**0.003**	**0.003**	**0.000**	0.974 (0.955–0.993)	**0.007**	**0.008**	**0.000**
C16:1	0.940 (0.906–0.975)	**0.001**	**0.001**	0.859	0.969 (0.942–0.997)	**0.031**	**0.041**	0.999
C18	1.027 (1.003–1.051)	**0.030**	**0.038**	0.865	1.042 (1.011–1.074)	**0.007**	**0.009**	0.689
C18:1	0.986 (0.974–0.997)	**0.017**	**0.011**	0.788	0.994 (0.984–1.005)	0.271	0.478	0.988
C18:2OH	0.994 (0.989–0.998)	**0.006**	**0.008**	0.546	0.995 (0.99–0.999)	**0.020**	**0.020**	0.625
**Metabolites**	**Diabetes + high risk CVD/ controls**				**Diabetes + high risk CVD/ diabetes**			
	OR (95% CI)	*P*-Value	*P*-Value^a^	*P*-Value^b^	OR (95% CI)	*P*-Value	*P*-Value^a^	*P*-Value^b^
Serine	1.023 (1.005–1.041)	**0.010**	**0.008**	**0.000**	1.002 (0.989–1.015)	0.732	0.630	0.341
C10:1	1.004 (1.001–1.08)	**0.037**	**0.027**	0.879	1.001 (0.997–1.005)	0.579	0.372	0.578
C14:1	0.983 (0.97–0.997)	**0.016**	**0.010**	**0.000**	1.005 (0.99–1.019)	0.534	0.631	0.072
C14:2	0.980 (0.965–0.994)	**0.006**	**0.005**	**0.000**	1.005 (0.99–1.021)	0.509	0.610	0.532
C16:1	0.976 (0.956–0.997)	**0.023**	**0.025**	0.742	1.039 (1.003–1.076)	**0.034**	**0.028**	**0.036**
C18	1.032 (1.008–1.056)	**0.008**	**0.009**	0.988	1.005 (0.982–1.029)	0.683	0.560	0.830
C18:1	0.989 (0.979–0.999)	**0.027**	0.060	0.854	1.003 (0.991–1.016)	0.590	0.311	0.060
C18:2OH	0.998 (0.996–1.0)	0.083	0.081	0.889	1.005 (1.0–1.009)	**0.043**	0.072	0.122

Results were shown as odds ratio (OR) and the corresponding 95% confidence intervals (CI)

^a^ Adjusted *P*-Value by age, BMI, and time of menopause

^b^ Adjusted *P*-Value by age, BMI, time of menopause, lipid profile, diabetes duration, and medications

C10:1, Decenoylcarnitine; C14:1, Tetradecenoylcarnitine; C14:2, Tetradecadienoylcarnitine; C16:1, Hexadecenoylcarnitine; C18, Octadecanoylcarnitine; C18:1, Octadecenoylcarnitine; C18:2-OH, 3-OH-octadecadienoyl

Entirely, acylcarnitine C18 and amino acid serine were significantly higher in diabetes, and acylcarnitines C14:1, C14:2, C16:1, C18:1, and C18:2OH were lower. In diabetes with a low risk of CVD vs control, serine, C10:1, and C18 were increased significantly and C14:2, C16:1, and C18:2OH acylcarnitines were decreased. In diabetes with a high risk of CVD vs control, increased acylcarnitines and amino acid were similar to diabetes with low-risk of CVD and C14:1, C14:2, C16:1, and C18:1 acylcarnitines were decreased. In diabetes with a low risk of CVD vs diabetes, there was no significantly changed metabolite. In diabetes with a high risk of CVD vs diabetes, C16:1 and C18:2OH were increased. There were no significant changes in other comparisons of study groups. These metabolites differed significantly within the compared groups after adjusting for age, BMI, and time of menopause. But after adding lipid profiles and medications to the adjustment model, just serine, C14:1, C14:2 remained significant.

**Fig. 2:**
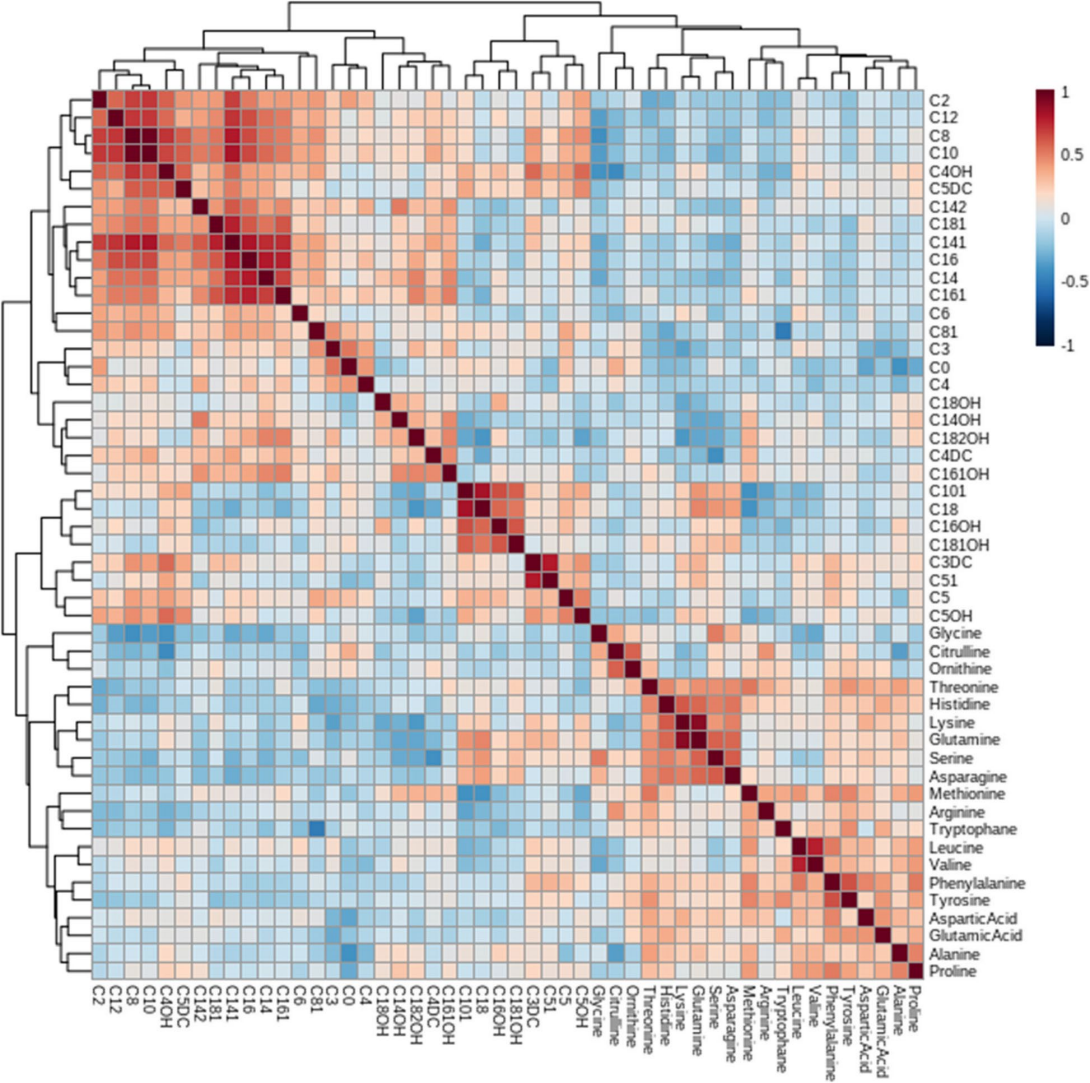
Correlation matrix showing the results of Pearson correlation analysis. Pearson correlation coefficient values and directions are marked with different colors; positive correlation (from white to red on the color scale); negative correlation (from white to blue) (see color-bar next to the matrix)

The heatmap represented the correlation between many significantly altered long-chain acylcarnitines that is presented in Fig. 2.

## Discussion

The present study provided a new avenue for investigating the correlations of acylcarnitine and amino acid metabolites with diabetes, and CVD risk among them. DM is an established risk factor for the development of CVD and people with DM have an increased risk of CVD complications compared with those with either condition alone. Various studies have also indicated different risks of CVD through gender between those with DM. In this study, 10 out of the 50 analyzed metabolites changed significantly that showed odds of diabetes and CVD may somewhat stem from dysregulation of serum acylcarnitines and amino acids profile.

### Acylcarnitines

Glucose, fatty acids, and amino acids are required substrates for energy generation and building blocks of macromolecules. Organs are adaptable in the selection of substrate which they will be used to sustain energy homeostasis. In the heart, liver, and skeletal muscle, the major metabolic pathway is fatty acid β-oxidation (FAO), which takes place in mitochondria [20].

The uptake of long-chain and very long-chain fatty acids into cells seems to be mediated by membrane proteins, called fatty acid transport proteins (FATPs). Although passive transport also occurs. After translocation across the plasma membrane, fatty acids are rapidly converted to acyl-CoAs at the cytosolic site. Acyl-CoAs converted to acylcarnitine by carnitine palmitoyltransferase 1 (CPT1) to import into mitochondria by carnitine acylcarnitine translocase (CACT). In the mitochondrial matrix, acylcarnitines reconverts into acyl-CoA by carnitine palmitoyltransferase 2 (CPT2). Subsequently, Acyl-CoAs break down into acetyl-CoA components through the chain of enzyme reactions, known as β-oxidation. The resultant acetyl-CoA then enters the mitochondrial tricarboxylic acid (TCA) cycle to produce more energy [21].

Disorders of the mitochondrial FAO pathway have been suggested to take part in the pathogenesis of diabetes and insulin resistance. Furthermore, FAO is an extremely important pathway in the heart while it continuously depends on fatty acids as a great proportion of its energy requirements. This coincides with the link of developing CVD in patients with DM [22].

As the major finding of this study, C14:1, C14:2, C16:1, C18, C18:1, and C18:2OH acylcarnitines were altered in diabetes compared to controls. Meanwhile, C10:1, C14:2, C16:1, and C18 metabolites changed similarly among diabetes with different severity of CVD compared to controls. As well, C16:1 and C18:2OH acylcarnitines increased in diabetes with a high risk of CVD compared to diabetes without CVD.

Some studies have indicated that metabolic profiles of acylcarnitines, mainly medium- and long-chain ones were associated with DM [23], with the risk of CVD in DM [4, 24], and also with CVD and mortality [25, 26]. There is a lack of agreement across the decreased or increased amount of specific short-, medium-, and long-chain acylcarnitines in association with diabetes incidence. Besides, reports concerning the fluctuations of FAO and TCA intermediates in diabetics are controversial [23, 27, 28]. As a notice, *Lu Y *et al. pointed out that the mitochondrial dysregulation caused by acylcarnitines may aggravate the development of DM more exactly than act as a trigger [29].

In terms of complications, a study conducted based on comparisons within diabetes and diabetes different complications, individuals with diabetic vascular complications showed down-regulation of some medium- and long-chain acylcarnitines [30]. As well, medium-chain (such as C10:1, C14:1, C16:1) and long-chain (such as C18, C18:1) acylcarnitines were positively associated with cardiovascular events such as mortality, death/ myocardial infarction in *Shah SH *et al. study [26]. Concordantly, we found similar trends for C10:1, 16:1, and C18 acylcarnitines especially in diabetes with a high risk of CVD while the results for C14:1 and C18:1 were in the opposite direction. Regarding the reduced amounts of acylcarnitines in this study, it was supposed that in patients experiencing a long-term period of the disease, the TCA cycle activity arises intending to supply energy for human bodies. Meanwhile, as the clinical period prolonged, diabetes metabolism would much get worse [30].

Contrary to these findings, *Zhao S *et al. study resulted in elevated C10, C12, C14:1, C14, C14-OH, and C16-OH which are mainly medium-chain acylcarnitines, among diabetes with coronary artery disease, heart failure, or stroke [4]. Based on the *Violante S *et al., the cause of this controversy might be; i) the variety of precursors that can generate medium-chain acylcarnitines, ii) their diverse functions in the FAO in the mitochondria [31], and iii) influence of diet on pathways related to acylcarnitine metabolism [32].

Despite this, studies concerning acylcarnitines in DM with/ without risk of CVD are still limited, and controversial discoveries have been stated.

### Amino acids

Regularly, body cells utilize glucose and lipids to provide the energy they need. However, they can use amino acids for energy supply, too [33]. There are more than twenty amino acids, each of which involves not the same degradation pathway. Nevertheless, amino acids mainly undergo shared reactions like deamination and transamination. Therefore, over these reactions all amino acids can be directly or through the production of pyruvate or acetyl-CoA transformed into mediates of TCA cycle and energy production [20].

The present study revealed that in diabetes vs control, the amino acid serine was increased significantly. In both diabetes with a low and high risk of CVD vs control, the serine was increased, too. In diabetes with low and high-risk CVD vs diabetes, there were no significantly changed amino acid metabolites.

As stated by *Lin w *et al., amino acid and fatty acid metabolism were the most involved pathways between diabetics and non-diabetic controls and serine was the focal point metabolite of diabetes [34]. More ever, *Walford GA *et al. identified the serine as a metabolite positively associated with incident diabetes [35]. A similar result was seen in animal studies, too [36] that provide other evidence in support of our results on the above amino acid in diabetics compared to controls. Whereas, Gunther S et al. reported that serine was inversely associated with diabetes risk [23].

Even though the exact reasons that led to our findings cannot be discovered according to the present study, possibilities obtained from the existing literature in this regard should be considered. The metabolic differences observed between baseline and post-treatment in pharmacometabolomic studies to some extent reveal the effects of medications on the altered metabolites. For instance, The profile of lipids and amino acids can be influenced by medications such as aspirin, statins, and antihypertensive drugs [37]. By this, confirming the predictive power of metabolites and clarifying their roles becomes a big challenge. So, it would be better to employ metabolomic approaches separately in the study of CVD predictors and treatment outcomes.

Ultimately, our findings together with others could provide further indications about metabolomics appliances in the prediction and diagnosis of diabetic postmenopausal women with CVD risk. Nonetheless, further studies with a larger patient population, and follow-ups are necessary to provide a more complete picture of metabolic profile and evaluation of novel biomarkers. Moreover, the influence of dietary assessment should be noted.

## Conclusion

In summary, our study described the associations of diabetes and its CVD risk with serum amino acids and acylcarnitines profile in postmenopausal women. The obtained in this study could be useful for understanding what might lead to DM or CAD and facile future prediction of these diseases.

## Supplementary Information

**Additional file 1: Figure S1** The result of data preprocessing after Cube root transformation and pareto scaling. **Figure S2** PLS-DA model validation by permutation tests based on separation distance. **Table S1** Measured concentrations of metabolites in 4 study groups. **Table S2** Measured coefficient of variation (%) of metabolites. **Table S3** Significantly altered metabolites among groups’ classification using one-way ANOVA analysis and Tukey’s HSD test.

## Data Availability

The datasets analysed during the current study are not publicly available due to ethical restrictions but are available from the corresponding author on reasonable request.

## Abbreviations

CVD: Cardiovascular disease
DM: Diabetes mellitus
CACS: Coronary artery calcium score
ACAD: Atherosclerotic coronary artery disease
ESI: Electrospray ionization
MRM: Multiple reaction monitoring
PCA: Principal component analysis
PLS-DA: Partial least squares discriminant analysis
VIP: Variable importance in the project
ROC: Receiver operating characteristi
AUC: Area under the curve
BMI: Body mass index
GFR: Low-density lipoprotein cholesterol
LDL-C: Glomerular filtration rate
HDL-C: High-density lipoprotein cholesterol
ALT: Alanine aminotransferase
AST: Aspartate aminotransferase
ACEi: Angiotensin-converting enzyme inhibitors
ARB: Angiotensin receptor blockers
CCB: Calcium channel blockers
C10:1: Decenoylcarnitine
C14:1: Tetradecenoylcarnitine
C14:2: Tetradecadienoylcarnitine
C16:1: Hexadecenoylcarnitine
C18: Octadecanoylcarnitine
C18:1: Octadecenoylcarnitine
FAO: 3-OH-octadecadienoylcarn, C18:2-OH, Fatty acid β-oxidation
FATPs: Fatty acid transport proteins
CPT1: Carnitine palmitoyltransferase
CACT: Carnitine acylcarnitine translocase
TCA: Tricarboxylic acid
