# Supplementary material for: Circulating amino acids and acylcarnitines correlated with different CAC score ranges in diabetic postmenopausal women using LC–MS/MS based metabolomics approach
Source: BMC Endocr Disord. 2022 Jul 21;22:186. doi: 10.1186/s12902-022-01073-9 (PMC9306187; doi:10.1186/s12902-022-01073-9)
Supplement: Supplementary file 1 — Additional file 1: Figure S1 The result of data preprocessing after Cube root transformation and pareto scaling. Figure S2 PLS-DA model validation by permutation tests based on separation distance. Table S1 Measured concentrations of metabolites in 4 study groups. Table S2 Measured coefficient of variation (%) of metabolites. Table S3 Significantly altered metabolites among groups’ classification using one-way ANOVA analysis and Tukey’s HSD test. [file 12902_2022_1073_MOESM1_ESM.docx]

**Supplementary Figure S1.** The result of data preprocessing after Cube root transformation and pareto scaling.


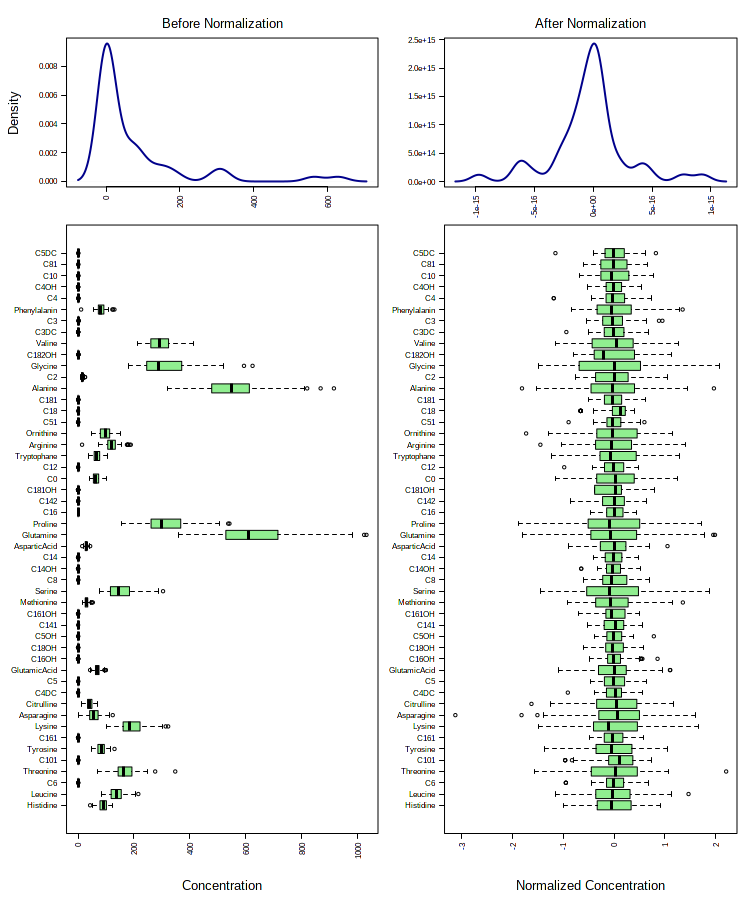


**Supplementary Figure S2.** PLS-DA model validation by permutation tests based on separation distance.


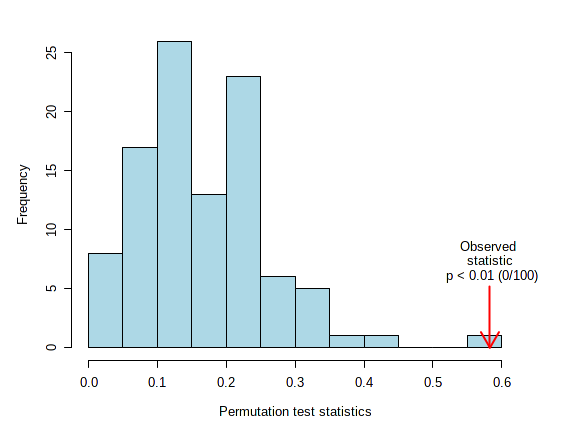


**Supplementary Table S1.** Measured concentrations of metabolites in 4 study groups.

| **Metabolites**  **(µmol/ L)** | **Controls** | | **Diabetes without CVD** | | **Diabetes+ Low-risk CVD** | | **Diabetes+ High-risk CVD** | |
| --- | --- | --- | --- | --- | --- | --- | --- | --- |
| **Alanine** | 582.195 | 138.407 | 553.331 | 103.637 | 542.991 | 118.051 | 578.955 | 98.853 |
| **Aspartic acid** | 28.780 | 4.890 | 30.460 | 6.124 | 28.374 | 5.146 | 29.316 | 4.185 |
| **Glutamic acid** | 70.159 | 9.983 | 71.331 | 9.159 | 69.855 | 18.752 | 68.975 | 10.141 |
| **Leucine** | 145.990 | 28.796 | 140.641 | 28.642 | 133.567 | 22.242 | 136.376 | 23.443 |
| **Methionine** | 33.577 | 7.874 | 29.653 | 5.527 | 29.823 | 5.946 | 29.442 | 6.731 |
| **Phenylalanine** | 83.045 | 12.514 | 89.287 | 15.618 | 82.601 | 13.953 | 80.855 | 10.415 |
| **Tyrosine** | 83.334 | 14.990 | 87.261 | 15.763 | 78.512 | 14.830 | 79.530 | 14.238 |
| **Valine** | 294.070 | 45.595 | 303.044 | 47.498 | 289.586 | 45.757 | 299.100 | 49.279 |
| **Arginine** | 123.877 | 20.291 | 119.533 | 19.406 | 126.812 | 29.738 | 117.742 | 20.880 |
| **Citrulline** | 41.236 | 11.034 | 41.943 | 14.117 | 45.640 | 12.549 | 39.406 | 11.121 |
| **Glycine** | 323.485 | 89.094 | 318.331 | 112.670 | 353.550 | 121.130 | 339.029 | 82.280 |
| **Ornithine** | 104.275 | 20.395 | 97.699 | 24.259 | 111.224 | 25.603 | 100.589 | 20.315 |
| **Proline** | 331.275 | 111.301 | 348.600 | 96.140 | 317.186 | 74.878 | 298.690 | 47.147 |
| **Threonine** | 173.319 | 41.388 | 175.072 | 36.685 | 187.150 | 67.973 | 166.966 | 34.704 |
| **Serine** | 127.261 | 32.254 | 161.501 | 59.586 | 174.658 | 71.150 | 167.293 | 36.307 |
| **Histidine** | 84.985 | 17.421 | 90.144 | 10.395 | 85.969 | 17.260 | 88.472 | 14.613 |
| **Lysine** | 183.750 | 52.505 | 207.125 | 36.820 | 195.536 | 39.239 | 194.452 | 42.297 |
| **Tryptophane** | 71.038 | 15.226 | 65.307 | 14.545 | 64.130 | 13.850 | 63.509 | 12.938 |
| **Asparagine** | 47.440 | 19.589 | 60.709 | 17.022 | 67.987 | 28.273 | 59.910 | 19.832 |
| **Glutamine** | 576.423 | 159.347 | 663.766 | 110.097 | 652.082 | 152.745 | 649.119 | 125.255 |
| **C0** | 65.047 | 15.628 | 62.356 | 11.382 | 64.900 | 9.623 | 68.747 | 11.872 |
| **C2** | 15.436 | 3.848 | 14.335 | 3.252 | 14.602 | 3.574 | 14.997 | 3.010 |
| **C3** | .947 | .272 | .845 | .179 | .881 | .195 | 1.037 | .366 |
| **C3DC** | .097 | .061 | .103 | .054 | .123 | .067 | .099 | .043 |
| **C4** | .456 | .142 | .364 | .081 | .430 | .148 | .446 | .188 |
| **C4OH** | .058 | .022 | .064 | .030 | .063 | .024 | .062 | .017 |
| **C4DC** | .077 | .024 | .052 | .019 | .057 | .024 | .058 | .018 |
| **C5** | .205 | .067 | .266 | .087 | .228 | .058 | .234 | .055 |
| **C5:1** | .062 | .030 | .077 | .034 | .079 | .038 | .064 | .022 |
| **C5OH** | .088 | .020 | .111 | .038 | .097 | .023 | .101 | .026 |
| **C5DC** | .300 | .089 | .322 | .162 | .320 | .139 | .295 | .080 |
| **C6** | .160 | .048 | .166 | .075 | .158 | .094 | .177 | .087 |
| **C8** | .231 | .102 | .209 | .144 | .255 | .166 | .213 | .096 |
| **C8:1** | .258 | .120 | .265 | .110 | .278 | .110 | .312 | .115 |
| **C10** | .306 | .151 | .247 | .181 | .305 | .220 | .247 | .115 |
| **C10:1** | .150 | .194 | .233 | .170 | .316 | .225 | .267 | .135 |
| **C12** | .122 | .044 | .098 | .044 | .113 | .053 | .104 | .037 |
| **C14** | .072 | .023 | .054 | .021 | .057 | .020 | .062 | .019 |
| **C14:1** | .148 | .054 | .098 | .050 | .119 | .060 | .107 | .036 |
| **C14:2** | .146 | .067 | .079 | .043 | .078 | .046 | .088 | .036 |
| **C14OH** | .030 | .014 | .014 | .005 | .015 | .006 | .018 | .009 |
| **C16** | .203 | .062 | .153 | .048 | .173 | .056 | .175 | .040 |
| **C16OH** | .021 | .026 | .013 | .008 | .018 | .009 | .028 | .028 |
| **C16:1OH** | .046 | .029 | .025 | .010 | .028 | .017 | .033 | .019 |
| **C16:1** | .086 | .040 | .041 | .019 | .054 | .021 | .059 | .029 |
| **C18** | .033 | .037 | .056 | .025 | .067 | .032 | .060 | .021 |
| **C18:1** | .213 | .082 | .149 | .059 | .180 | .084 | .159 | .047 |
| **C18OH** | .042 | .023 | .017 | .007 | .019 | .008 | .023 | .015 |
| **C18:1OH** | .015 | .028 | .013 | .007 | .019 | .011 | .028 | .030 |
| **C18:2OH** | .472 | .318 | .102 | .136 | .125 | .138 | .275 | .349 |

Results reported as mean and standard deviation (SD)

**Supplementary Table S2.** Measured coefficient of variation (%) of metabolites.

| **Metabolites**  **(µmol/L)** | **QC level 1** | | **QC level 2** | | **HMDB-ID** |
| --- | --- | --- | --- | --- | --- |
|  | **Intra assay (CV%)** | **Interassay (CV%)** | **Intra assay (CV%)** | **Interassay (CV%)** |  |
| **Alanine** | 1.9 | 3.4 | 2.2 | 4.8 | 0000161 |
| **Aspartic acid** | 8.5 | 11.7 | 2.5 | 4.8 | 0000191 |
| **Glutamic acid** | 2.9 | 4.9 | 2.9 | 5 | 0000148 |
| **Leucine** | 1.4 | 10.1 | 2.5 | 6.2 | 0000687 |
| **Methionine** | 2.7 | 11.8 | 3.5 | 12.1 | 0000696 |
| **Phenylalanine** | 1.9 | 9.7 | 3 | 5.8 | 0000159 |
| **Tyrosine** | 2.15 | 5.2 | 2.4 | 5.5 | 0000158 |
| **Valine** | 1.8 | 4.2 | 1.7 | 6.1 | 0000883 |
| **Arginine** | 4 | 7.7 | 3 | 8.1 | 0000517 |
| **Citrulline** | 3.52 | 10.6 | 3.4 | 7.3 | 0000904 |
| **Glycine** | 1.6 | 4.9 | 1.7 | 4.9 | 0000123 |
| **Ornithine** | 2.7 | 3.4 | 2.4 | 5.7 | 0000214 |
| **Proline** | 1.8 | 4.7 | 2.2 | 6 | 0000162 |
| **Threonine** | 8.2 | 5.9 | 9.2 | 8.9 | 0000167 |
| **Serine** | 9.3 | 8.7 | 9.1 | 7.6 | 0000187 |
| **Histidine** | 14.8 | 15 | 13.2 | 9.1 | 0000177 |
| **Lysine** | 10.9 | 14.3 | 9.6 | 12.5 | 0000182 |
| **Tryptophane** | 6.5 | 11.5 | 4.4 | 10.2 | 0000929 |
| **Asparagine** | 10.9 | 16.2 | 8.4 | 16.1 | 0000168 |
| **Glutamine** | 3.9 | 10.7 | 8.4 | 9.6 | 0000641 |
| **C0** | 4.9 | 7.9 | 4.5 | 7.7 | 0000062 |
| **C2** | 5.9 | 12.5 | 5.3 | 8.4 | 0000201 |
| **C3** | 5.4 | 11.3 | 5.2 | 8.4 | 0000824 |
| **C3DC** | 5.8 | 10.5 | 5.4 | 8.5 | 0002095 |
| **C4** | 7.7 | 11.7 | 4.1 | 8.7 | 0002013 |
| **C4OH** | 8.4 | 11.6 | 5.8 | 8.2 | 0000736 |
| **C4DC** | 7.4 | 11.7 | 7.9 | 8.4 | 0061717 |
| **C5** | 6.6 | 11.5 | 7.5 | 8.4 | 0000688 |
| **C5:1** | 6.5 | 11.4 | 7.2 | 8.3 | 0002366 |
| **C5OH** | 6.4 | 11.7 | 7.2 | 8.4 | 0061189 |
| **C5DC** | 6.7 | 13.5 | 6.1 | 8.5 | 0013130 |
| **C6** | 6.6 | 12.3 | 4.0 | 9.2 | 0000756 |
| **C8** | 5.9 | 14.3 | 5.9 | 7.8 | 0000791 |
| **C8:1** | 6.1 | 13.8 | 6.1 | 7.9 | 0013324 |
| **C10** | 8.2 | 17.0 | 6.6 | 10.5 | 0000651 |
| **C10:1** | 8.5 | 17.5 | 6.4 | 10.8 | 0013205 |
| **C12** | 7.3 | 15.2 | 6.1 | 13.3 | 0002250 |
| **C14** | 6.3 | 14.8 | 6.2 | 15.2 | 0005066 |
| **C14:1** | 6.5 | 14.9 | 5.8 | 14.8 | 0002014 |
| **C14:2** | 6.2 | 14.2 | 5.4 | 14.5 | 0240756 |
| **C14OH** | 6.7 | 14.6 | 5.9 | 15.0 | 0061640 |
| **C16** | 6.3 | 9.5 | 4.0 | 10.8 | 0240774 |
| **C16OH** | 7.1 | 10.2 | 4.2 | 11.0 | 0013336 |
| **C16:1OH** | 7.3 | 9.9 | 4.5 | 10.1 | 0013333 |
| **C16:1** | 6.8 | 9.5 | 4.4 | 9.8 | 0013207 |
| **C18** | 5.2 | 15.5 | 5.3 | 13.0 | 0000848 |
| **C18:1** | 5.4 | 14.9 | 5.5 | 12.8 | 0094687 |
| **C18OH** | 5.3 | 14.7 | 5.3 | 12.5 | 0013154 |
| **C18:1OH** | 5.2 | 15.2 | 5.3 | 13.2 | 0013339 |
| **C18:2OH** | 6.1 | 15.5 | 5.5 | 13.4 | 0240780 |

**Supplementary Table S3.** Significantly altered metabolites among groups’ classification using one-way ANOVA analysis and Tukey’s HSD test.

| **Name** | **P-value** | **Post-hoc (Tukey’s HSD)** |
| --- | --- | --- |
| Serine | 0.019 | Diabetes-Control; Diabetes+ High-Risk CVD-Control; Diabetes+ Low-Risk CVD-Control |
| C10:1 | 0.009 | Diabetes+ High-Risk CVD-Control; Diabetes+ Low-Risk CVD-Control |
| C14:1 | 0.018 | Diabetes-Control; Diabetes+ High-Risk CVD-Control |
| C14:2 | 0.016 | Diabetes-Control; Diabetes+ High-Risk CVD-Control; Diabetes+ Low-Risk CVD-Control |
| C16:1 | 0.000 | Diabetes-Control; Diabetes+ High-Risk CVD-Control; Diabetes+ Low-Risk CVD-Control; Diabetes+ High-Risk CVD-Diabetes |
| C18 | 0.000 | Diabetes-Control; Diabetes+ High-Risk CVD-Control; Diabetes+ Low-Risk CVD-Control |
| C18:1 | 0.026 | Diabetes-Control; Diabetes+ High-Risk CVD-Control |
| C18:2OH | 0.005 | Diabetes-Control; Diabetes+ Low-Risk CVD-Control; Diabetes+ High-Risk CVD-Diabetes |
